# Investigating the Correlation Between Cognitive Function and Fasting Blood Sugar, Fasting Insulin Level and Insulin Sensitivity in Patients With Multiple Sclerosis

**DOI:** 10.1002/edm2.70006

**Published:** 2024-10-07

**Authors:** Nasim Rezaeimanesh, Naghme Abbasi Kasbi, Roghayyeh Saeedi, Mohammad Ali Sahraian, Soodeh Razeghi Jahromi, Abdorreza Naser Moghadasi

**Affiliations:** ^1^ Multiple Sclerosis Research Center Neuroscience Institute, Tehran University of Medical Sciences Tehran Iran

**Keywords:** cognitive function, fasting blood sugar, insulin, multiple sclerosis

## Abstract

**Introduction:**

There has been a surge in interest in identifying the factors that impact cognitive impairment (CI) in patients with multiple sclerosis (MS). The purpose of our study was to examine the correlation between fasting blood sugar (FBS), fasting insulin level, as well as insulin sensitivity and cognitive function in patients with MS.

**Material and Methods:**

A total of 85 patients with MS enrolled in this cross‐sectional study. Insulin sensitivity (IS) was determined using the quantitative insulin sensitivity check index (Quicki) formula. Cognitive function was evaluated using the Persian version of the Brief International Cognitive Assessment for MS (BICAMS). Spearman correlation test was employed to examine the correlation between cognition and FBS, insulin and IS.

**Results:**

The mean ± SD age of the participants was 39.4 ± 10.2 years, and 62 (72.9%) were female. The participants had a FBS level of 87.05 ± 11.73 mg/dL, insulin level of 10.14 ± 7.57 μU/mL and a Quicki index of 0.36 ± 0.05. A higher score on the BVMT‐R and BVMT‐R‐Delayed subtests showed a significant negative correlation with FBS (*r*: −0.32; *p*: 0.003 and *r*: −0.31; *p*: 0.004, respectively). Conversely, a significant negative correlation (*r*: −0.24; *p*: 0.031) was observed between higher fasting insulin levels and the CVLT_II score. IS showed a positive correlation with the CVLT‐II (*r*: 0.24; *p*: 0.027) and BVMT_R (*r*: 0.21; *p*: 0.054) subtests.

**Conclusion:**

Our data indicate that elevated fasting glucose, developed fasting insulin levels and reduced insulin sensitivity may serve as potential predictors for CI in patients with MS.

## Graphical abstract

Higher fasting blood sugar, insulin level and lower insulin sensitivity are correlated with lower cognitive performance in patients with multiple sclerosis.

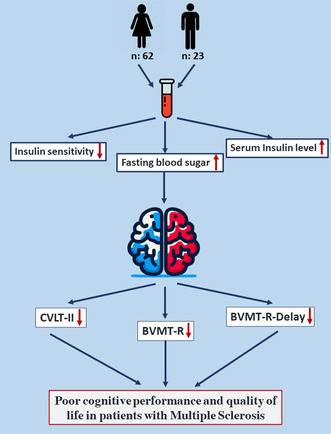

## 1 Introduction

Cognitive psychology explains a wide range of cognitive processes identified for neuropsychological evaluation, including perception, memory, language, reasoning, problem‐solving and decision‐making [1]. Cognitive impairment (CI) is prevalent among patients with multiple sclerosis (MS), affecting a substantial proportion (40%–60%) of these patients. Common areas of cognitive dysfunction include sustained attention, verbal fluency, recent memory, visuospatial perception and conceptual reasoning. These challenges can impact routine activities, work and individual's quality of life [2].

The prevalence of MS has shown an increase globally, including Iran. It is estimated that around 2.8 million people worldwide are currently living with MS, resulting in a rate of 35.9 cases per 100,000 population [3]. MS prevalence is reported 167.54 cases per 100,000 subjects in Tehran, the capital city of Iran in 2020 [4]. Among adults, MS is the most common neuroinflammatory disease, with a higher impact on individuals aged 20 to 40 years [5]. It can also reduce life expectancy by 7.5 years compared to the general population [6].

The CI associated with MS have an impact on various aspects of life, including household management, social participation and employment maintenance, all of which contribute to the patient's overall quality of life [7]. Various factors such as older age, younger age at disease onset and higher levels of disability have been found to be associated with CI in patients with MS. Other potentially modifiable risk factors include physical exercise, mentally active lifestyles, comorbidities, vitamin D levels, smoking, alcohol, pain and sleep abnormalities [8]. Additionally, our previous study has documented a potential association between elevated serum LDL cholesterol and total cholesterol levels, and cognitive dysfunction among individuals with MS [9].

Despite advancements, there still exists an ongoing concern regarding the management of cognitive dysfunction in patients with MS, since there are currently no approved medications specifically targeting cognitive symptoms in MS. Recent studies have emphasised the probable link between metabolic disorders and CI [10]. Research has suggested that insulin plays a crucial role in maintaining proper brain function. Abnormalities in peripheral insulin levels can increase the risk of memory loss and neurodegeneration. Consequently, insulin resistance (IR) could exacerbate phenomena common to both MS and insulin‐resistant conditions, such as CI and heightened inflammatory reactions [11]. The results of a recent systematic review on the relation between IR and cognitive function highlighted the association between IR and reduced verbal and numerical reasoning skills, along with diminished processing speed [12].

Identifying and addressing modifiable factors that contribute to CI in patients with MS holds immense value and can significantly improve their overall quality of life. Limited studies have mentioned fasting blood sugar, fasting insulin, insulin sensitivity and their correlation with cognitive dysfunction in patients with MS. The current study aims to evaluate the correlation between fasting blood sugar, fasting insulin, insulin sensitivity and CI in patients affected by various forms of MS. Investigating this correlation can lead to finding metabolic disorders that can be modified by lifestyle changes or drug therapy, and this improvement can ultimately lead to a betterment in the cognitive ability of patients with MS.

## 2 Materials and Methods

### 2.1 Study Participants

A cross‐sectional investigation was conducted at the MS specialist clinic of Sina Hospital in Tehran, Iran, spanning from December 2021 to September 2022. A total of 85 patients with MS, drawn from those who visited the clinic during the study period, expressed interest in participating, met the inclusion criteria and were consequently enrolled in the study.

The definitive diagnosis of MS was established in accordance with the revised McDonald criteria of 2017 [13] and validated by neurologist confirmation.

Inclusion criteria encompassed individuals aged between 18 and 55 years, an Expanded Disability Status Scale (EDSS) score of less than 5.5, an educational background of at least a diploma, absence of other neurological disorders besides MS and no concurrent chronic illnesses such as gastrointestinal, liver, kidney, heart, respiratory, hypertension, diabetes mellitus or cancer‐related conditions. Additionally, not to be undergoing treatment for depression, not following any specific diet, more than 1 month since their last corticosteroid pulse therapy or relapse, and not to be pregnant or lactating constituted further inclusion criteria.

### 2.2 Patient's Consent and Protocol Approval

Study aims and method was explained for all participants, and written consent was obtained. Study protocol was reviewed and approved in the ethics committee of Tehran University of Medical Science by the ethic number: IR.TUMS.NI.REC.1400.039.

### 2.3 Data Collection

Demographic and clinical information were obtained through a personal information form administered in face‐to‐face interviews. The EDSS was determined by a skilled neurologist during the examination of patients. Measurements of weight and height were taken using a Seka scale, with minimal clothing, and a tape measure, with accuracies of 100 g and 0.5 cm, respectively. Body mass index (BMI) was computed using the formula weight (kg)/ [height (m)]^2^.

The assessment of physical activity level utilised by the international physical activity questionnaire short form (IPAQ‐SF) [14]. The metabolic equivalents of tasks (MET)‐minute/week were computed based on the frequency and duration of physical activity by assigning MET values to each category (walking: 3.3 METs, moderate activity: 4.0 METs, vigorous activity: 8.0 METs) [15]. MET was considered as a continuous variable in analysis, and a higher score showed a higher physical activity.

Participants' cognitive function was evaluated using the Persian version of the Brief International Cognitive Assessment for Multiple Sclerosis (BICAMS) at the base of investigation. BICAMS subtests were validated for Iranian patients with MS in 2012 [16]. BICAMS consists of three subtests: the California Verbal Learning Test second edition (CVLT‐II), the Symbol Digit Modalities Test (SDMT) and the Brief Visuospatial Memory Test‐Revised (BVMT‐R). Additionally, there are two delayed tests specifically for CVLT‐II and BVMT‐R. The CVLT‐II subtest is designed to assess deficits in verbal learning and memory. The SDMT subtest is employed to evaluate processing and motor speed, while the BVMT‐R is utilised to assess visuospatial learning. The ranges of BICAMS subtests scoring are as follow: CVLT‐II: 0–80, SDMT: 0–110, BVMT‐R: 0–36, CVLT‐II‐delay: 0–16 and BVMT‐R‐delay: 0–12 [16]. We considered each subtest score as a continuous variable, which a higher score indicated a higher ability in cognitive function.

At the study's onset, a 5 cc blood sample was taken from the antecubital vein of all participants following a minimum overnight fasting period of 10 h. Fasting insulin level and FBS were determined by enzymatic colorimetric assay. Insulin sensitivity (IS) based on quantitative insulin sensitivity check index (Quicki) was calculated using [ISQUICKI = 1/(log (fasting insulin (mU/L)) + log (fasting glucose (mg/dL)))] formula [17]. QUICKI is an effective index for evaluating insulin sensitivity in humans. Previous studies have demonstrated that QUICKI exhibits correlation with insulin sensitivity measures derived from the hyperinsulinemic‐euglycemic clamp in both diabetic and nondiabetic individuals [18].

### 2.4 Statistical Analysis

The data were processed using SPSS 26 software, and the normal distribution of variables was assessed through the Kolmogorov–Smirnov test. Numerical variables were presented as mean ± SD, and categorical variables were reported as number (percentage). The correlation between FBS, IS, insulin and BICAMS subtests was checked using Spearman correlation test. *p*‐value less than 0.05 was considered as significant level. The optimal cut‐off points for QUICKI were considered 0.34 in men and 0.33 in women [19].

### 2.5 Sample Size Calculation

Sample size was calculated 80 subjects using the below formula, by considering *r* : 0.31 [9], power: 80% and 95% confidence level.

$$n={\left(Z_{\alpha /2}+Z_{\beta }/C\;\right)}^{2}+3$$

## 3 Results

A total of 85 individuals diagnosed with MS were enrolled in the current investigation. Table 1 presents the baseline characteristics of the study participants.

**TABLE 1:** Baseline characteristics of participants.

Variables		
Female gender^a^		62 (72.9%)
Age (years)^b^		39.4 (10.2)
EDSS^b^		3.0 (1.7)
Annual relapse rate (number) ^$^		0.7 (1.4)
Disease duration (years) ^$^		10.8 (5.8)
Body mass index (kg/m^2^)		24.9 (4.8)
Metabolic equivalent of tasks (MET‐min/wk)		918.9 (1359.4)
MS type^a^		
RRMS		53 (62.4%)
SPMS		28 (32.9%)
PPMS		4 (4.7%)
Treatment^a^		
Ocrelizumab		55 (64.7%)
Tysabri		25 (29.4%)
Rituximab		1 (1.2%)
Fingolimod		3 (3.5%)
Dimethyl fumarate		1 (1.2%)
Marital status^a^		
Single		22 (25.9%)
Married		57 (67.1%)
Divorced		3 (3.5%)
Death of wife/ husband		3 (3.5%)
Education^a^		
Diploma		29 (34.1%)
Associate degree		10 (11.8%)
Bachelor		33 (38.8%)
Master of sciences		10 (11.8%)
PhD or higher		3 (3.5%)
Employment status^a^		
Employment		43 (50.6%)
Unemployment		42 (49.4%)
Cigarette smoker^a^		
Yes		16 (18.8%)
No		69 (81.2%)
Alcohol consumer^a^		
Yes		16 (18.8%)
No		69 (81.2%)
FBS^c^ (mg/dL)		84.00 (12.75)
Serum insulin^c^ (μU/mL)		8.80 (10.05)
IS^c^ (Quicki index)		0.34 (0.07)

*Note:* Annual attack rate for patients with RRMS.

Abbreviations: EDSS, Expanded Disability Status Scale; FBS, fasting blood sugar; IS, insulin sensitivity; PPMS, primary progressive MS; RRMS, relapsing–remitting MS; Serum insulin, fasting insulin levels; SPMS, secondary progressive MS.

^a^ These data are presented as number (percent).

^b^ These data are presented as mean (standard deviation).

^c^ These data are presented as median (IQR).

The participants exhibited an average age of 39.4 ± 10.2 years, and 72.9% (*n*: 62) of them were female. The mean ± SD values for the EDSS, annual attack rate and disease duration among patients were 3.0 ± 1.7, 0.7 ± 1.4 and 10.8 ± 5.8 years, respectively. The study participants demonstrated a mean ± SD BMI of 24.9 ± 4.8 kg/m^2^ and a MET of 918.9 ± 1359.4 MET‐min/week. Of the patient population, 62.4% (*n*: 53) were diagnosed with relapsing–remitting MS (RRMS), 32.9% (*n*: 28) presented with secondary progressive MS (SPMS), while the remaining individuals exhibited the primary progressive form of the disease (PPMS). The majority of participants were undergoing Ocrelizumab treatment (*n*: 55; 64.7%), followed by Tysabri (*n*: 25; 29.4%), Fingolimod (*n*: 3; 3.5%), Rituximab (*n*: 1; 1.2%) and Dimethyl fumarate (*n*: 1; 1.2%). Among the participants, 57 (67.1%) individuals were married, while 22 (25.9%) were single. Regarding educational attainment, 38.8% (*n*: 33) held a bachelor's degree, and 34.1% (*n*: 29) possessed a diploma. In terms of employment status, 42 (49.4%) of the patients with MS were unemployed, with 78.5% (*n*: 33) of them being homemakers. Sixteen participants (18.8%) were cigarette smokers, mirroring the exact count of individuals who consumed alcohol.

The median (IQR) levels among participants for FBS, insulin and the Quicki index were 84.00 (12.75) mg/dL, 8.80 (10.05) μU/mL and 0.34 (0.07), respectively.

Table 2 reports the median levels of BICAMS subtest results and compares the scores between two groups of insulin sensitive and insulin resistance participants. The median (IQR) amount of BICAMS subtests was as follow: CVLT‐II = 47.00 (15.00); SDMT = 39.00 (21.00); BVMT‐R = 23.00 (14.50); CVLT‐II‐delay = 10.00 (4.00); and BVMT‐R‐delay = 10.00 (5.50).

**TABLE 2:** The scores of BICAMS subtests in total participants and by insulin sensitivity and resistance.

Variables		Total *n*: 85	Insulin sensitive *n*: 61	Insulin resistance *n*: 24	*p*
BICAMS subtests	CVLT‐II	47.00 (15.00)	48.00 (13.25)	40.00 (15.5)	0.066
	SDMT	39.00 (21.00)	37.50 (23.25)	40.50 (25.50)	0.714
	BVMT‐R	23.00 (14.50)	24.00 (12.00)	19.50 (16.50)	**0.045**
	CVLT‐II‐delayed recall	10.00 (4.00)	10.00 (3.00)	9.00 (6.00)	0.163
	BVMT‐R‐Delayed recall	10.00 (5.50)	10.00 (4.00)	9.00 (8.50)	0.069

*Note:* All data are presented as median (Interquartile range). *p*‐values are calculated using Mann–Whitney test. Bold values present significant *p* values.

Abbreviations: BICAMS, Brief International Cognitive Assessment for MS; BVMT‐R, Brief Visuospatial Memory Test‐Revised; CVLT‐II, California Verbal Learning Test second edition; SDMT, Symbol Digit Modalities Test.

The median level of BVMT‐R subtest was significantly higher in insulin sensitive group compared with insulin resistance group (24.00 (12.00) vs. 19.50 (16.50); *p*: 0.045).

**FIGURE 1:**
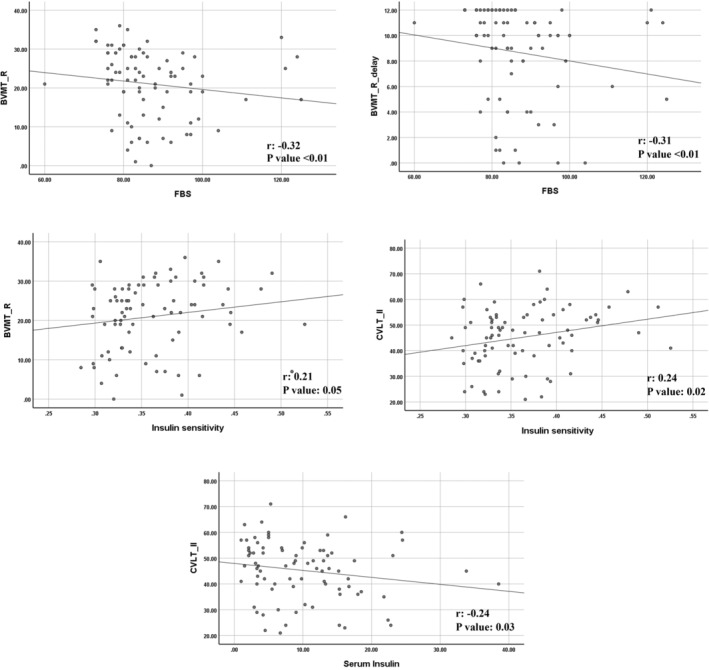
The correlations between subtests of Brief International Cognitive Assessment for Multiple Sclerosis (BICAMS) and FBS, fasting serum insulin and insulin sensitivity. FBS: Fasting blood sugar; Serum insulin shows the fasting levels of insulin; Insulin sensitivity is calculated based on quantitative insulin sensitivity check index (Quicki). *p*‐values are calculated using Spearman correlation test. BVMT‐R: Brief Visuospatial Memory Test‐Revised; CVLT‐II: California Verbal Learning Test second edition.

Table 3 and Figure 1 illustrate the correlation among FBS, fasting insulin levels, IS and BICAMS subtests. A significant inverse correlation was founded between levels of FBS and BVMT‐R (*r*: −0.32; *p*: 0.003), as well as BVMT‐R‐delay (*r*: −0.31; *p*: 0.004) subtests of BICAMS. IS based on Quicki index showed a direct correlation with cognitive function of patients with MS. So that, the results of Spearman correlation test were (*r*: 0.24; *p*: 0.027) for IS and CVLT‐II, and (*r*: 0.21; *p*: 0.054) for IS and BVMT‐R. On the contrary, fasting serum insulin levels indicated a significant inverse correlation with CVLT‐II (*r*: −0.24; *p*: 0.031). The results of other correlation tests were not significant (*p* > 0.05).

**TABLE 3:** The correlation between fasting blood sugar (FBS), fasting insulin levels, insulin sensitivity (IS) and BICAMS subtests.

	CVLT‐II	SDMT	CVLT‐delayed recall	BVMT‐R	BVMT‐ R‐delayed recall
FBS	−0.06 (0.547)	−0.15 (0.162)	−0.18 (0.108)	**−0.32 (0.003)**	**−0.31 (0.004)**
Insulin	**−0.24 (0.031)**	0.03 (0.784)	−0.10 (0.345)	−0.19 (0.080)	−0.18 (0.093)
IS (Quicki index)	**0.24 (0.027)**	−0.02 (0.798)	0.11 (0.306)	**0.21 (0.054)**	0.20 (0.072)

*Note:* Data are presented as *r* (*p*‐value), calculated by Spearman correlation test. Bold values present significant *p* values.

Abbreviations: BICAMS, Brief International Cognitive Assessment for MS; BVMT‐R, Brief Visuospatial Memory Test‐Revised; CVLT‐II, California Verbal Learning Test second edition; FBS, Fasting blood sugar; Insulin, fasting insulin levels; IS, insulin sensitivity; SDMT, Symbol Digit Modalities Test.

## 4 Discussion

The current investigation represents the examination of the correlation between FBS, fasting insulin, and IS, with CI in patients with MS. The study's findings indicate that better performance on the BVMT‐R and its delayed recall subtests is associated with lower FBS levels. Additionally, higher fasting insulin levels were linked to poorer scores on the CVLT‐II. Insulin sensitivity showed a positive correlation with performance on both the CVLT‐II and BVMT‐R subtests, suggesting a notable connection between metabolic factors and cognitive function in patients with MS.

Previously, we reported possible correlation between the increased serum LDL and total cholesterol as another metabolic parameter, and CI among patients with MS [9]. Existing studies on the role of blood sugar on CI have primarily focused on the correlation between insulin and its sensitivity with cognitive dysfunction [20]. Therefore, one strength of this study is that it represents a comprehensive examination of the correlation between whole FBS, IS and fasting insulin level, and cognitive dysfunction in patients with MS for the first time.

Our findings align with previous research comparing cognitive performance in 74 relapsing–remitting patients with MS, stratified by the presence of IR. Cognitive assessments including the CVLT, Controlled Oral Word Association Test and Judgement of Line Orientation Test indicated significantly reduced performance in insulin‐resistant patients with MS. Furthermore, an inverse relationship was observed between insulin levels and cognitive scores, highlighting pronounced verbal memory and spatial comprehension deficits in this subgroup [20].

Cognitive dysfunction associated with MS predominantly affects recent memory, information processing speed, sustained attention and executive functions [21]. Emerging evidence underscores a strong relation between metabolic dysregulation and neurodegenerative pathways. Key hormones including leptin, ghrelin, insulin and insulin‐like growth factor 1 (IGF‐1) are implicated in modulating neuronal injury, mitigating neurotoxic challenges and influencing various neurodegenerative mechanisms [22]. Notably, reduced serum levels of IGF‐1 have been correlated with CI and fatigue in patients with MS [23].

Prior literature has demonstrated that IR constitutes a significant risk factor for cognitive dysfunction, as well as neurodegenerative conditions such as Alzheimer's disease (AD) and Parkinson's disease (PD) [24]. The role of insulin in cerebral glucose metabolism, peptide regulation, neurotransmitter modulation and inflammatory processes is suggested mechanisms through which IR may influence neurodegenerative pathways [11].

The pathophysiology of MS is marked by the intricate interplay of immunological and neuronal factors, leading to complex and multifaceted biological mechanisms underlying MS‐related CI. The pathology of MS and consequent CI is linked to various pathways including inflammation, demyelination and subsequent remyelination, oxidative stress, blood–brain barrier dysfunction, viral antigenic effects and cellular metabolism disruptions [25]. Structural and functional disruptions in neuronal networks due to inflammation are variably associated with CI. Evidence has correlated biomarkers of axonal damage, such as cerebrospinal fluid neurofilament light chain (CSF NfL), with decrements in information processing speed, implicating white matter lesion burden and cortico‐subcortical disconnection as contributing factors. Notably, neuroinflammation characterised by the aberrant activation of glial elements within the central nervous system (CNS) has been associated with attenuated attention and information processing speed [26].

The inflammatory aetiology of MS appears to precipitate hyperinsulinemia and elevate the risk of IR [27]. Disruption of immune and inflammatory homeostasis is considered a primary driver of MS pathology. A contributing factor to the elevated incidence of IR in patients with MS may be an imbalance between activated inflammatory mediators and the quantity and functionality of Th17 cells and regulatory T cells (Tregs). An increased frequency of T cells in patients has the potential to induce an upsurge of inflammatory cytokines within CNS lesions. These inflammatory mediators, by interfering with metabolic processes, may dysregulate insulin signalling pathways, thereby promoting insulin resistance [28]. Insulin resistance can precipitate hyperglycaemia. Hyperglycaemia is recognised for its pro‐inflammatory characteristics, stimulating an acute immune response characterised by the enrolment of inflammatory mediators including leukocytes and cytokines, integral to glucose metabolism. This acute inflammatory response to hyperglycaemia can lead to systemic inflammation and subsequent neuropathy [29]. Both localised and systemic inflammation are implicated in the breakdown of the blood–brain barrier (BBB), impaired waste clearance and enhanced immune cell infiltration. Such pathological changes may result in the disruption of glial and neuronal cell integrity, leading to hormonal imbalances, heightened immune reactivity or CI. The inflammatory sequelae of metabolic syndrome are associated with the pathogenesis of neurodegenerative diseases [15].

Thus, MS may exacerbate insulin resistance, further elevating blood glucose levels. Conversely, increased blood glucose can intensify inflammatory processes, thereby elevating the risk of cognitive impairment. This bidirectional relationship underscores the intertwined nature of metabolic, inflammatory and neurodegenerative processes in the context of MS which can lead to cognitive deficits.

These results provide further support for the hypothesis that metabolic parameters can affect cognitive functions in patients with MS.

The limitation of the current study is the lack of measurement of inflammatory factors as mediators of the relationship between impaired sugar and insulin metabolism and cognitive function in patients with MS.

## 5 Conclusion

Our findings suggest that heightened fasting glucose levels, increased fasting insulin concentrations and diminished insulin sensitivity could function as potential prognostic markers for cognitive impairment in patients with MS. Interventions such as lifestyle modifications or pharmacotherapy may be efficacious in managing these metabolic parameters. These observations underscore the importance of further investigative efforts to enhance cognitive outcomes and the broader quality of life for individuals with the diagnosis of MS.

## 6 Limitation and Strengths

Cognitive dysfunction is a wide prevalent problem in patients with MS which could affects their quality of life. We investigated modifiable factors correlated with this problem, and it is valuable point of view in the aim of this study. However, even though QUICKI is a validated and practical method for estimating insulin sensitivity and resistance it is not the gold standard, and this is our limitation. We suggest conducting studies with a larger sample size and in the form of a cohort for a more detailed investigation of metabolic disorders as a cause of cognitive impairment in patients with MS.

## Author Contributions

**Nasim Rezaeimanesh:** data collection, data entry and analysis, drafting the manuscript and approving the final format of the manuscript. **Naghme Abbasi Kasbi, Roghayyeh Saeedi, Soodeh Razeghi Jahromi** and **Mohammad Ali Sahraian:** data collection, drafting the manuscript and approving the final format of the manuscript. **Abdorreza Naser Moghadasi:** hypothesis, supervision, data collection, drafting the manuscript and approving the final format of the manuscript.

## Conflicts of Interest

Mohammad Ali Sahraian and Abdorreza Naser Moghadasi have received educational, research grants, lecture honorarium, travel supports to attend scientific meetings from Biogen‐Idec, Merck‐Serono, Cinnagen, Zistdaru, Zahravi and Genzyme. The other authors declare no conflicts of interest.

## Data Availability

The data that support the findings of this study are available from the corresponding author upon reasonable request.
